# Supplementary material for: ﻿Phylogenomics reveal Populusgonggaensis as a hybrid between P.lasiocarpa and P.cathayana (Salicaceae)
Source: PhytoKeys. 2024 Jan 23;237:161–77. doi: 10.3897/phytokeys.237.103012 (PMC10829108; doi:10.3897/phytokeys.237.103012)
Supplement: Supplementary material 1 — Information of taxa that were not used for phylogenetic analysis in Populussubg.Tacamahaca [file phytokeys-237-161_article-103012__-s001.doc]

**Phylogenomics reveal *Populus gonggaensis* as a hybrid between *P. lasiocarpa* and *P. cathayana* (Salicaceae)**

Wenyan Du^1^, Yachao Wang^3^, Dajun Xie^2^, Enze Li^1^, Yuran Bai^1^, Ce Shang^1^, Zhixiang Zhang^1^

^1^ *Laboratory of Systematic Evolution and Biogeography of Woody Plants, School of Ecology and Nature Conservation, Beijing Forestry University, Beijing 100083, China*

^2^ *Sichuan Academy of Forestry, Chengdu 610000, China*

^3^ *School of Life Science, Fudan University, Shanghai 200433, China*

Corresponding author: Ce Shang (ce_shang@bjfu.edu.cn) and Zhixiang Zhang (zxzhang@bjfu.edu.cn)

**Abstract**

High levels of intra-specific polymorphism and frequent hybridisation make it difficult to define species and correctly apply their scientific names. *Populus* L. is a challenging genus with plentiful natural and artificial hybrids. This study is a part of the project ‘Flora of Pan-Himalaya’ and aims to determine the taxonomic identity of *P. gonggaensis* N. Chao & J.R. He and to find out whether it is of hybrid origin. Whole-genome sequencing data were obtained from 57 samples. The SNP matrix was developed for phylogenetic reconstruction, ABBA-BABA statistics, PCA and ADMIXTURE analysis. The results indicate that *P. gonggaensis* is a spontaneous hybrid between *P. lasiocarpa* and *P. cathayana*. This study points out the importance of SNP data and comprehensive analyses for discovering the potential interspecific hybridisation and clarifies the usage of the name. In addition, the lectotype of *P. gonggaensis* was designated.

**Keywords**

*Populus gonggaensis*, whole genome resequencing, hybrid origin

**Introduction**

The genus *Populus* L. (Salicaceae), embraces ca. 60 tree species that are widely distributed and cultivated throughout the Northern Hemisphere (Dickmann 2001; POWO 2023). Most of the species of this genus play an important role in temperate forest ecosystems and numerous cultivars have arisen through hybridisation and artificial selection (Eckenwalder 1996).

According to morphological features, *Populus* was divided into six sections, i.e. sect. *Abaso* Eckenw., sect. *Turanga* Bunge, sect. *Populus* (= sect. *Leuce* Duby.), sect. *Leucoides* Spach, sect. *Aigeiros* Duby and sect. *Tacamahaca* Spach (Eckenwalder 1996). However, phylogenetic studies, based on nucleotide sequences, showed different results. Both nuclear and plastid sequences inferred that sect. *Tacamahaca* and sect. *Aigeiros* are polyphyletic (Yun et al. 2015; Liu et al. 2017). Using genome-wide nuclear single nucleotide polymorphism and chloroplast genome data, Wang et al. (2022) reconstructed the phylogeny of genus *Populus* and suggested a classification that recognises four subgenera, subg. *Abaso* (Eckenw.) C. Shang, Y.C. Wang & Z.X. Zhang, subg. *Turanga* (Bunge) Dode, subg. *Populus* and subg. *Tacamahaca* (Spach) Kamelin (= subg. *Eupopulus* Dode, *nom. inval.*; containing species of sect. *Leucoides*, sect. *Aigeiros* and sect. *Tacamahaca*). The topology of the main clades of *Populus* is relatively clear; however, the phylogenetic and taxonomic positions of certain species remain problematic. High levels of morphological variation and reticulate evolution have led to a highly controversial species delimitation (Eckenwalder 1996). For instance, Fang et al. (1999) recorded 71 *Populus* species distributed within China, but Eckenwalder (1996) recognised only 29 species all over the world.

Hybridisation occurs amongst species of *Populus* and many species have been proved to be of hybrid origin. When published, *P. wulianensis* S.B. Liang & X.W. Li and *P. ningshanica* Z. Wang & S.L. Tung were regarded as species, but an integrative study, based on molecular evidence and morphological analysis, revealed both of them as hybrid species (Zhang et al. 2018; Liu et al. 2022). *Populus × irtyschensis* Chang Y. Yang was also shown to be a filial generation whose parents should be *P. nigra* L. and *P. laurifolia* Ledeb. (Feng et al. 2013; Shang et al. 2016). Recently, on the basis of whole-genome sequencing, Liu et al. (2022) examined 227 individuals from 12 taxa of the sect. *Populus*, recognised seven species and identified five hybrid taxa. For the genus *Populus*, interspecific hybridisation poses an obstacle to species delimitation and their possible hybrid origin (Belyaeva 2020). Therefore, it is important to conduct studies on some problematic taxa with potential hybridisation phenomena.

High-resolution molecular markers and comprehensive analysis are required in identification of potential hybridisation. Microsatellite (SSR) or a few nuclear/plastid sequences are mostly not enough to provide sufficient informative sites to detect complicated relationships resulting from reticulate evolution (Shang et al. 2022). The first sequenced woody plant genome was *P. trichocarpa* Torr. & A. Gray ex Hook. (Tuskan et al. 2006), a synonym of the earlier name, *P*. *tristis* Fisch., which is the correct name and currently accepted (Skvortsov 2010; Belyaeva et al. 2020, POWO 2023) and should be used in scientific papers instead of the redundant *P*. *trichocarpa*. With the continuous development of sequencing technology, whole genome sequence data have been increasingly published and whole-genome resequencing technology could provide sufficient single nucleotide polymorphism (SNP) to deal with taxonomic problems (Hirota et al. 2022). With unique superiority, whole-genome resequencing has also been widely used in phylogenetic and evolutionary studies of *Populus* (Wang et al. 2020; Liu et al. 2022; Wang et al. 2022), especially in detecting hybrids and exploring reticulate evolution.

*Populus gonggaensis* N. Chao & J.R. He has been described, based on specimens collected from the eastern margin of the Tibetan Plateau (Chao 1991). Several natural *Populus* species are sympatric in the vicinity of where *P. gonggaensis* was found, including two species from sect. *Leucoides*, i.e. *P. lasiocarpa* Oliv. and *P. glauca* Haines [= *P*. *wilsonii* C.K. Schneid. (Skvortsov 2008; Shang 2017)] and a number of species from sect. *Tacamahaca*, i.e. *P. cathayana* Rehder, *P. trinervis* Z. Wang & S.L. Tung, *P. szechuanica* C.K. Schneid., *P. rockii* (Rehder) H.L. Yang and *P. xiangchengensis* Z. Wang & S.L. Tun (Fang 1985; Fang et al. 1999).

With lobed flower disc, *P. gonggaensis* was thought to be a close relative of *P. lasiocarpa* or *P. glauca* and placed in sect. *Leucoides*. However, hairs on the surface of the abaxial leaf veins and branchlets are relatively short and procumbent, which differ from the long and twisted hairs of *P. lasiocarpa* and *P. glauca*. However, *P. gonggaensis* shows similarity in these characters with species of the sect. *Tacamahaca*.

Shang (2017) argued that *P. gonggaensis* should be treated as an ambiguous species, which needs further research, due to the fact that no wild individuals have been seen since the collection of the type specimen. The follow-up phylogenetic studies on *Populus* species have not taken *P. gonggaensis* into consideration (Wang et al. 2020; Wang et al. 2022). Unfortunately, no more specimens, which conform to the description in the original paper of *P. gonggaensis* or are similar to the type specimen, have been found either in the herbaria collections or digital collections available. Due to the large-scale development of industry in modern China, the locality where the type specimen was collected, has been turned into a newly-built area of Kangding. We also have not found plants that could be identified as *P. gonggaensis* in our recent fieldwork. Thus, it is important to conduct systematic research on *P. gonggaensis*, which can also provide phylogenetic evidence for species definition and taxonomic revision of *Populus* in the future.

**Materials and Methods**

**Sample collection and sequencing**

A total of 57 samples represent 22 species including three species from sect. *Leucoides* and almost all species of subg. *Tacamahaca* distributed in China (Table 1). Only taxa of the subg. *Tacamahaca* occurring in the *P. gonggaensis* area were included in the study, excluded taxa being summarised in Table S1. The samples of *P. gonggaensis* were obtained from one of the syntypes stored at the Herbarium of Sichuan Academy of Forestry (SCFI). For other taxa, fresh leaves were collected from adult trees and dried in silica gel. Voucher specimens were deposited at the Herbarium of Beijing Forestry University (BJFC). Previous whole-genome resequencing was also downloaded from the National Center for Biotechnology Information (NCBI) database and the BIG Data Center, Beijing Institute of Genomics (BIG), Chinese Academy of Sciences.

We used the CTAB method with minor modifications to extract the whole-genomic DNA from leaf samples (Doyle and Doyle 1987). All DNA samples were shipped to BerryGenomics (China) for subsequent sequencing. Whole-genome paired-ends reads with a target coverage of 10× were generated using Illumina NovaSeq 6000 platform (Illumina, San Diego, CA, United States).

**Read mapping and SNP calling**

Nuclear variants were discovered with BWA, SAM tools and GATK tools. First, the resequencing data for each sample was mapped to the reference genome of *P. trichocarpa* (Tuskan et al. 2006) using the default parameters of BWA-MEM v.0.7.17-r1188 (Li and Durbin 2009). Then, the mapped reads were converted to BAM files and sorted and filtered using the SAMtools package v.1.6 (Li et al. 2009). PCR duplications were marked using the Picard tool v.2.1.1. We used GATK v.4.1.4 (McKenna et al. 2010) with HaplotypeCaller to call a single sample of short variants and GATK with CombineGVCFs to combine all samples of short variants. SNPs were called using the SelectVariants tool implemented in GATK. Filters implemented in GATK were applied to the SNPs with the parameters as "QD < 10.0 || FS > 60.0 || MQ < 40.0 || SOR > 3.0 || MQRankSum < -12.5 || ReadPosRankSum < -8.0" (Danecek et al. 2011). Next, the depth of each SNP was counted and the average depth was calculated. Finally, using GATK, the SNP dataset was generated by filtering by depth the minor allele frequencies. Finally, the “-st” command was used to specify the model when the data used were for DNA.

**Phylogenetic Analyses**

Python v.2.7.5 was used to convert SNPs into phylip format and IQ-tree v.2.0.3 to analyse the dataset (Felsenstein 1993; Nguyen et al. 2015). We used “-alrt” to specify that the number of repetitions of SH-aLRT branch test was 1000 (Guindon et al. 2010). A Maximum Likelihood (ML) phylogenetic tree was constructed by IQ-tree under the most appropriate model selected by ModelFinder (Kalyaanamoorthy et al. 2017). *Populus euphratica* was selected as the outgroup.

**PCA and ADMIXTURE Analysis**

PLINK v.1.9.0 (Purcell et al. 2007) was used for LD-based SNP filtering. A principal component analysis (PCA) of screened species of *Populus* was performed with PLINK, based on whole genome SNPs and graphs were built using the ‘ggplot2’ package (Wickham 2016) in R. ADMIXTURE software (Alexander et al. 2009) was used for Maximum Likelihood estimation of individual ancestors from multi-locus SNP genotype datasets. Moreover, admixture uses a fast numerical optimisation algorithm that allows for faster calculation of estimates. Then, we used ADMIXTURE v.1.3.0 to study the population structure of some individuals and the number of clusters (K) was set from 1 to 10. Finally, the optimal K value was selected by cross-validation. The cross-validation used by ADMIXTURE is to divide the genotype data into several parts, use one part as the test set and the rest as the training set and then calculate the log-likelihood value. The times of cross-validation can be specified by the -cv=n parameter, where n is the number of splits. ADMIXTURE will output the cross-validation error (CV error). The K value is the most appropriate (most ideal species and population number) when the cross-validation error value is at its lowest.

**ABBA-BABA Statistics**

To detect gene flow from other species into *P. gonggaensis*, we performed ABBA-BABA statistics to calculate gene flow from potential parents. ABBA-BABA Statistics (also known as D-statistics) provided a model to calculate deviations from a strictly bifurcated evolutionary history using genome-scale SNP data, in order to test for gene penetrance (Martin et al. 2015; Malinsky et al. 2021). Briefly, the relationship amongst three populations and an outgroup was assumed to be (((P_1_, P_2_), P_3_), O) and this model could test whether there was an excess of shared variation between P_2_ and P_3_ compared to that between P_1_ and P_3_ (Durand et al. 2011). The D-value was the ratio of the difference in the number of ABBA sites and BABA sites to the sum of the two types of sites. The larger the value of D, the stronger the degree of gene flow from P_2_ to P_3_. If the absolute value of the Z-score was higher than 3, it would be considered statistically significant (Busing et al. 1999). D software was used for gene flow analysis. The vcf file containing the SNP dataset was imported, a directory was created and the outgroup was specified.

**Results**

**Sequence Data Processing**

We collected and performed whole genome resequencing for nine individuals sampled from *P. heterophylla*, *P. glauca* and *P. haoana*, with an average depth of 10× for each individual. In total, 735.14 GB of clean data of 57 individuals were obtained for single nucleotide polymorphism (SNP) calling. Clean data were mapped against the *P. trichocarpa* reference genome and strict analyses, 4,790,248 high-quality SNPs were obtained. The total SNP dataset was used for all analyses.

**Phylogeny based on SNP**

The ML tree was built using the total SNP dataset obtained, with *P. euphratica* set as the outgroup and the other 21 species clustered into three clades (Fig. 1A). Two sect. *Leucoides* species, *P. heterophylla* Du Roi and *P. glauca* Haines were firstly divergent. The second clade included some species of the sect. *Tacamahaca* and sect. *Aigeiros*, i.e. *P. trinervis, P. kangdingensis* Z. Wang & S.L. Tung*, P. qamdoensis* Z. Wang & S.L. Tung*, P. yunnanensis* Dode*, P. simonii* Carrière*, P. iliensis* Drobow (= *P*. *usbekistanica* Kom.)*, P. nigra* L. and *P. afghanica* (Aitch. & Hemsl.) C.K. Schneid. (= ***P. nigra***). *Populus gonggaensis* and *P. lasiocarpa* (sect. *Leucoides*) formed a monophyletic clade, which was sister of other species of sect. *Tacamahaca* represented by *P. cathayana* and *P. szechuanica*. The bootstrap values of all the interspecies nodes in this tree were extremely high.

**PCA Analysis**

To facilitate the observation of the results, the data of the outgroup (*P. euphratica*) were removed from the SNP dataset for PCA analysis. Individuals of sect. *Tacamahaca* were clustered together (upper left corner of the Fig. 2A), while individuals of sect. *Leucoides* were clustered in the lower right corner of the Figure (*P. heterophylla* is the North American-distributed species, which is the right-most off-centre point in Fig. 2A). *P. gonggaensis* was located between the two sections. Combining the ML tree and the result of PCA analysis that involved species that occur in the area within or around the type locality of *P. gonggaensis*, we selected the 12 species closest to *P. gonggaensis* in each of the two sections in the PCA, which include *P. glauca*, *P. cathayana, P. koreana, P. lasiocarpa, P. rockii, P. laurifolia, P. ciliata, P. pseudoglauca, P. szechuanica, P. haoana* and *P. xiangchengensis.* Species of sect. *Tacamahaca* still gathered, but *P. lasiocarpa* became separated from *P. glauca* (Fig. 2B). Fig. 2C was plotted by adding PC3 (Z-axis) to Fig. 2B. Individuals in the blue circles include all samples from *P. cathayana*, and *P. koreana*, while samples in the red circles were *P. lasiocarpa*. *P. gonggaensis* is located between *P. lasiocarpa* and several species of sect. *Tacamahaca* (*P. cathayana* and *P. koreana*)

**ADMIXTURE Analysis**

The SNP dataset for admixture analysis covered 38 individuals of 12 species, amongst which 37 samples were the same as the 37 samples in PCA (Fig. 2B). Besides, *P. euphratica* was added as the outgroup to facilitate the calculation and accuracy for the visualisation of the dataset. The population structure was analysed using K values from 1 to 15 and the optimal K value was calculated as 9 (Fig. 3B). Each of the two assumed parents, *P. lasiocarpa* and *P. cathayana*, was recognised as an independent species separated from other samples (Fig. 3B). Additionally, *P. gonggaensis* was an admixture of these two species.

**ABBA-BABA Statistics**

SNP datasets of 12 species were used for Dsuite calculations. Using *P. euphratica* as the outgroup, the gene flow was calculated for all trios and two result files (BBAA.txt and Dmin.txt) were generated, reacting to the introgression between P2 and P3 for each trio and containing all combinations with positive D-values after random operations, with the same P2-P3 species corresponding to different D-values depending on the P1 species. The results are shown in Fig. 3A. When *P. gonggaensis* is selected as P3 and the D value is positive, there are a total of 36 combination forms. Amongst the combinations that satisfy the condition, the species that could be located at P2 are *P. cathayana*, *P. koreana*, *P. lasiocarpa*, *P. ciliata*, *P. rockii*, *P. pseudoglauca*, *P. szechuanica* and *P. xiangchengensis*. The formula is used to calculate the results of all negative D-values associated with these eight species and the final data of total D-values are presented in the heat map in Fig. 3A. *Populus cathayana* has the highest D-value amongst all species and when it is P2, all the eight values are positive (Fig. 3A). This means that *P. cathayana* provides more genetic swaps with *P. gonggaensis* than any other species. Hence, the largest proportion of gene flow in *P. gonggaensis* is from *P. cathayana*. When *P. lasiocarpa* is P2, the ratio of gene flow to *P. gonggaensis* is only lower than that caused by *P. cathayana* and all other D-values are positive (Fig. 3A). Thus, *P. cathayana* and *P. lasiocarpa* are the species that swap the most genes with *P. gonggaensis*.

**Taxonomic treatment**

***Populus* × *gonggaensis*** N. Chao & J.R. He in Sichuan Forest. Sci. Techn. 12(3): 1, f. 1. 1991.

Type: China, Sichuan, Kangding County, Simaqiao, 2700 m elev., 27 May 1991, Neng Chao & Jiaren He 4207 (Lectotype in SCFI!, designated here; isolectotypes in SCFI!).

= ***Populus cathayana*** Rehder × ***Populus lasiocarpa*** Oliv.

There were four specimens of ‘Neng Chao & Jiaren He 4207’ found in SCFI and all of them were labelled as ‘TYPUS’. According to Art. 9.6 of ICN (Turland et al. 2018), the four specimens should be syntypes (Fig. S2). Hence, we designated the best-preserved one as the lectotype of *P*. × *gonggaensis* (Fig. 6).

**Discussion**

When published, *P. gonggaensis* was considered as a species that belongs to sect. *Leucoides*, according to morphological characteristics, such as deeply-lobed discs, tomentose leaves and pubescent capsules (Chao 1991). We observed the type specimens and found *P. gonggaensis* shows similarities in both *P. cathayana* and *P. lasiocarpa*. For example, the persistent floral discs on the fruit of *P. gonggaensis* are parted, which is similar to those of *P. lasiocarpa*. On the other hand, the leaves of *P. gonggaensis* are abaxially glabrous, which is similar to those of *P. cathayana*. Additionally, the morphology of *P. gonggaensis* is partly intermediate between that of its parents (Table 2, Fig. 4). Apart from the type specimens, not a single specimen could be indubitably identified as *P. gonggaensis,* based on morphological features. During the field survey, we did not find an individual which is consistent with the original description and protologue. The type of *P. gonggaensis* was collected from Simaqiao, which has now become an urban built-up area of Kangding City. *P. cathayana* has a wide distribution in the area and constitutes a sympatric species with *P. gonggaensis*.

According to the phylogenetic tree, *P. gonggaensis* is clustered with *P. lasiocarpa* with high support, which suggested a close relationship between them. However, the topology differed significantly from another phylogenetic research on the genus *Populus* (Wang et al. 2022). Whether species of sect. *Leucoides* formed a monophyletic clade, *P. lasiocarpa*, *P. glauca* and *P. heterophylla* are always basal taxa of subg. *Tacamahaca* (= subg*. Eupopul*us*, nom. inva*l.; Wang et al. (2020); Wang et al. (2022)). In this study, *P. gonggaensis* and *P. lasiocarpa* are clustered in the clade which is composed of sect. *Tacamahaca* and sect. *Aigeiros,* but set apart from *P. heterophylla* and *P. glauca* of sect. *Leucoides*. The introduction of hybrid individuals will alter the topology of the phylogenetic tree (Debray et al. 2022). Almost all the species have been analysed in earlier phylogenetic research (Wang et al. 2020, 2022), except for *P. gonggaensis*. Thus, when we removed *P. gonggaensis* data from the SNP matrix and reconstructed the ML tree, the topology was totally different (Fig. 1B). Simultaneously, we have reconstructed a species tree, which also solved this problem (Fig. S1). *Populus lasiocarpa* no longer clustered with the branches of sect. *Tacamahaca* and sect. *Aigeiros,* but clustered with *P. heterophylla* as a monophyly and all species of sect. *Leucoides,* located at the base of the tree. The PCA analysis showed that *P. gonggaensis* may be an intersectional hybrid and one of the parents could be *P. lasiocarpa*.

Results of ABBA-BABA analysis show that, when *P. lasiocarpa* is P2, the D value is < 0 only compared with *P. cathayana*, so it is more possible that *P. lasiocarpa* is the other parent rather than the remaining *Populus* species. In Fig. 3, when *P. koreana* is P2, the D values of *P. koreana* and *P. lasiocarpa* seem to be close; but when compared with *P. lasiocarpa*, the D value of *P. koreana* is smaller than that of *P. lasiocarpa*. Thus, the probability that *P. koreana* is the other parent is lower than that for *P. lasiocarpa.* There were two other reasons that can also rule out the possibility of *P. koreana* being a parent: firstly, *P. koreana* is distributed in north-eastern China and there is a very large geographical distance from where *P. gonggaensis* is distributed, which means there is no distribution overlap between *P. koreana* and *P. gonggaensis* (we speculate that the high contribution of *P. koreana* is due to the fact that it has a large portion of the gene flow of *P. cathayana* and that they may constitute a complex as found in previous phylogenetic studies); second, what we can find in Fig. 3A is that when *P. cathayana* is P2, the smallest D value occurs when *P. lasiocarpa* is P1, while the D value of *P. cathayana* is still considerable when *P. koreana* is P1 and *P. cathayana* is P2. Therefore, we believe that the D value is close to 0 when P1 and P2 were the two parents of P3, because gene flows occur between *P. gonggaensis* as P3 and both parents. These results suggest that *P. cathayana* and *P. lasiocarpa* are two potential parents for *P. gonggaensis*.

Our PCA study showed that *P. gonggaensis* may be an intersectional hybrid and one of the parents is *P. lasiocarpa* of sect. *Leucoides*, while the contribution of gene flow from *P. koreana* to *P. gonggaensis* is much lower, so the possibility of its being another parent is excluded. Finally, the ADMIXTURE result indicated that P. *gonggaensis* contains nearly equal components of both species, namely *P. cathayana* and *P. lasiocarpa* (Fig. 3B).

In conclusion, multiple methods provided evidence for a supposition that *P. gonggaensis* is a spontaneous hybrid between *P. lasiocarpa* and *P. cathayana*. During our field investigation, not a single individual matching the type specimens was discovered. In addition, *P. gonggaensis* is not a taxon, but a solitary hybrid individual, probably F1, which no longer occurs in the area from which it was described.

**Acknowledgements**

We thank the curators of SCFI for providing the necessary material. We are also grateful to the reviewers and the editor for constructive comments and suggestions that helped us to improve the manuscript. This work was financially supported by National Natural Science Foundation of China (grant no. 32001247) and the Second Tibetan Plateau Scientific Expedition and Research Program (grant no. 2019QZKK05020205).

**References**

Alexander DH, Novembre J, Lange K (2009) Fast model-based estimation of ancestry in unrelated individuals. Genome research 19: 1655–1664.

Belyaeva IV (2020). Challenges in identification and naming: Salicaceae *sensu stricto*. Skvortsovia: International Journal of Salicology and Plant Biology, 5(3), 83–104.

Belyaeva IV, Dutton C, Govaerts RHA, Liesebach H, McGinn K, Taylor G, Pickett J (2020). Verification of names for certain *Populus* L. clones (Salicaceae) commonly grown in the United Kingdom. Skvortsovia: International Journal of Salicology and Plant Biology, 6(3): 87–116.

Busing FM, Meijer E, Leeden RVD (1999) Delete-m jackknife for unequal m. Statistics and Computing 9: 3–8.

Chao N (1991) New taxa of sect. *Leucoides* Spach of gen. *Populu*s L. Journal of Sichuan Forestry Science and Technology 12(3): 1–3.

Chao N, Liu J, Gong G (2009) On the classification and distribution of the subfamily Populoideae (Salicaceae). Journal of Wuhan Botanical Research 27: 23–40.

Danecek P, Auton A, Abecasis G, Albers CA, Banks E, DePristo MA, Handsaker RE, Lunter G, Marth GT, Sherry ST (2011) The variant call format and VCFtools. Bioinformatics 27: 2156–2158.

Debray K, Le Paslier M-C, Bérard A, Thouroude T, Michel G, Marie-Magdelaine J, Bruneau A, Foucher F, Malécot V (2022) Unveiling the Patterns of Reticulated Evolutionary Processes with Phylogenomics: Hybridization and Polyploidy in the Genus *Rosa*. Mandel J (Ed.). Systematic Biology 71: 547–569. https://doi.org/10.1093/sysbio/syab064

Dickmann D (2001) Poplar culture in north America. NRC Research Press.

Doyle JJ, Doyle JL (1987) A rapid DNA isolation procedure for small quantities of fresh leaf tissue.

Durand EY, Patterson N, Reich D, Slatkin M (2011) Testing for ancient admixture between closely related populations. Molecular biology and evolution 28: 2239–2252.

Eckenwalder JE (1996) Systematics and evolution of *Populus*. Biology of *Populus* and its implications for management and conservation 7: 32.

Fang W (1985) Salicaceae. In: Editorial Board of 'Flora of Sichuan', Flora of Sichuan, vol. 3. Sichuan Publishing House of Science & Technology, Chengdu: 38–143

Fang ZF, Zhao SD, Skvortsov AK (1999) Salicaceae. In: Wu ZY, Raven PH (Eds.) Flora of China, vol. 4. Science Press, Beijing & Missouri Botanical Garden, St. Louis: 139–274.

Felsenstein J (1993) PHYLIP (phylogeny inference package), version 3.5 c. Joseph Felsenstein.

Feng J, Jiang D, Shang H, Dong M, Wang G, He X, Zhao C, Mao K (2013) Barcoding poplars (*Populus* L.) from western China. PLoS One 8: e71710.

Guindon S, Dufayard J-F, Lefort V, Anisimova M, Hordijk W, Gascuel O (2010) New algorithms and methods to estimate maximum-likelihood phylogenies: assessing the performance of PhyML 3.0. Systematic biology 59: 307–321.

Hirota SK, Yahara T, Fuse K, Sato H, Tagane S, Fujii S, Minamitani T, Suyama Y (2022) Molecular phylogeny and taxonomy of the Hydrangea serrata complex (Hydrangeaceae) in western Japan, including a new subspecies of H. acuminata from Yakushima. PhytoKeys 188: 49–71. https://doi.org/10.3897/phytokeys.188.64259

Kalyaanamoorthy S, Minh BQ, Wong TK, Von Haeseler A, Jermiin LS (2017) ModelFinder: fast model selection for accurate phylogenetic estimates. Nature methods 14: 587–589.

Li H, Durbin R (2009) Fast and accurate short read alignment with Burrows–Wheeler transforms. bioinformatics 25: 1754–1760.

Li H, Handsaker B, Wysoker A, Fennell T, Ruan J, Homer N, Marth G, Abecasis G, Durbin R (2009) The sequence alignment/map format and SAMtools. Bioinformatics 25: 2078–2079.

Liu X, Wang Z, Shao W, Ye Z, Zhang J (2017) Phylogenetic and taxonomic status analyses of the *Abaso* section from multiple nuclear genes and plastid fragments reveal new insights into the North America origin of *Populus* (Salicaceae). Frontiers in plant science 7: 2022.

Liu, S., Zhang, L., Sang, Y., Lai, Q., Zhang, X., Jia, C., ... & Wang, J. (2022). Demographic history and natural selection shape patterns of deleterious mutation load and barriers to introgression across *Populus* genome. Molecular biology and evolution, 39(2), msac008.

Malinsky M, Matschiner M, Svardal H (2021) Dsuite-Fast D-statistics and related admixture evidence from VCF files. Molecular Ecology Resources 21: 584–595.

Martin SH, Davey JW, Jiggins CD (2015) Evaluating the use of ABBA–BABA statistics to locate introgressed loci. Molecular biology and evolution 32: 244–257.

McKenna A, Hanna M, Banks E, Sivachenko A, Cibulskis K, Kernytsky A, Garimella K, Altshuler D, Gabriel S, Daly M (2010) The Genome Analysis Toolkit: a MapReduce framework for analyzing next-generation DNA sequencing data. Genome research 20: 1297–1303.

Nguyen L-T, Schmidt HA, Von Haeseler A, Minh BQ (2015) IQ-TREE: a fast and effective stochastic algorithm for estimating maximum-likelihood phylogenies. Molecular biology and evolution 32: 268–274.

Purcell S, Neale B, Todd-Brown K, Thomas L, Ferreira MA, Bender D, Maller J, Sklar P, De Bakker PI, Daly MJ (2007) PLINK: a tool set for whole-genome association and population-based linkage analyses. The American journal of human genetics 81: 559–575.

POWO (2023) Plants of the World Online. Facilitated by the Royal Botanic Gardens, Kew. Published on the Internet; http://www.plantsoftheworldonline.org/ [Accessed 11.08.2023].

Shang C, Zhang L, Zhang Z (2016) New combinations for nothotaxa of *Populus* (Salicaceae) from China. Phytotaxa 253: 176–178.

Shang C (2017) Taxonomic Review of *Populus* Linnaeus from Pan-Himalaya. PhD Thesis, Beijing Forestry University, China.

Shang C, Li E, Yu Z, Lian M, Chen Z, Liu K, Xu L, Tong Z, Wang M, Dong W (2022) Chloroplast Genomic Resources and Genetic Divergence of Endangered Species *Bretschneidera sinensis* (Bretschneideraceae). Frontiers in Ecology and Evolution 10: 873100. https://doi.org/10.3389/fevo.2022.873100

Skvortsov, A.K. (2008) The genus *Populus* L. (Salicaceae) of Indian Himalaya. Novosti Sistematiki Vysshikh Rastenii 40: 52-67.

Skvortsov AK (2010) Taxonomical synopsis of the genus *Populus* L. in East Europe, North and Central Asia. Byulleten' Glavnogo Botaniceskogo Sada 196: 62-73.

Turland NJ, Wiersema JH, Barrie FR, Greuter W, Hawksworth DL, Herendeen PS, Knapp S, Kusber W-H, Li D-Z, Marhold K, May TW, McNeill J, Monro AM, Prado J, Price MJ, Smith GF (2018) International Code of Nomenclature for algae, fungi, and plants (Shenzhen Code) adopted by the Nineteenth International Botanical Congress Shenzhen, China, July 2017. Regnum Vegetabile 159. Koeltz Botanical Books, Glashütten. https://doi.org/10.12705/Code.2018

Tuskan, G. A., Difazio, S., Jansson, S., Bohlmann, J., Grigoriev, I., Hellsten, U., Rokhsar, D. (2006) The genome of black cottonwood, Populus trichocarpa (Torr. & Gray). science, 313(5793), 1596-1604.

Wang M, Zhang L, Zhang Z, Li M, Wang D, Zhang X, Xi Z, Keefover‐Ring K, Smart LB, DiFazio SP, Olson MS, Yin T, Liu J, Ma T (2020) Phylogenomics of the genus *Populus* reveals extensive interspecific gene flow and balancing selection. New Phytologist 225: 1370–1382. https://doi.org/10.1111/nph.16215

Wang Y, Huang J, Li E, Xu S, Zhan Z, Zhang X, Yang Z, Guo F, Liu K, Liu D, Shen X, Shang C, Zhang Z (2022) Phylogenomics and Biogeography of *Populus* Based on Comprehensive Sampling Reveal Deep-Level Relationships and Multiple Intercontinental Dispersals. Frontiers in Plant Science 13: 813177. https://doi.org/10.3389/fpls.2022.813177

Wickham H (2016) ggplot2: Elegant Graphics for Data Analysis. Springer-Verlag New York. ISBN 978-3-319-24277-4

Yun T, Li JM, Zhou AP, Yan LX, Zong D, Li D, He CZ (2015) Analysis of Phylogenetic Relationship of *Populus* Based on Sequence Data of Chloroplast Regions. Plant Physiology Joumal 51(8): 1339–1346. doi: 10.13592/j.cnkippj.2015.0118

Zhang L, Wang M, Ma T, Liu J (2018) Taxonomic status of *Populus wulianensis* and P. ningshanica (Salicaceae). PhytoKeys 108: 117–129. https://doi.org/10.3897/phytokeys.108.25600

**Others**

**Table 1.** Summary of the statistics of genome resequencing data for 57 individuals of 21 species and one outgroup.

**Table 2.** Morphological comparison of *Populus gonggaensis* with *P. cathayana* and *P. lasiocarpa.*

**Figure 1.** (A) ML tree of 57 samples of the genus *Populus* reconstructed by IQ-TREE, based on 4,790,248 high-quality SNPs with an outgroup of *P. euphratica*; (B) ML tree of 56 samples (*P. gonggaensis* is deleted) of the genus *Populus* reconstructed by IQ-TREE, based on 4,790,248 high-quality SNPs with an outgroup of *P. euphratica*.

**Figure 2.** Principal Component Analysis (PCA), based on genetic distance using SNPs data. (A) All 57 samples representing 22 species; (B) 37 samples representing 12 species, including *Poppulus gonggaensis* and its most closely-related species; (C) Plotted by adding PC3 (Z-axis) to (B). Points inside red circle represents *P. lasiocarpa*, while those inside blue circle represent *P. cathayana* and *P. koreana*.

**Figure 3.** Hybrid introgression analysis using SNP matrix of *Populus* species. (A) The results of ABBA-BABA statistics which measured gene flow amongst 12 species when P3 = *P. gonggaensis*. When D > 0 and the D value is further away from 0, it indicates that a gene swap or hybridisation event is more likely to have occurred, which means the genes of P3 is more likely to swap with those of P1 or P2; (B) Population structure analysis for 12 species of *Populus* (K = 9). Each coloured bar represents one individual and coloured segments represent proportions of ancestral components. The number of individuals and species names for each lineage are shown at the bottom.

**Figure 4.** Morphological comparison of *Populus gonggaensis* with *P. cathayana* and *P. lasiocarpa*. (A) male flower of *P. cathayana* (floral disc entire); (B) female flower of *P. cathayana* (floral disc entire, ovary glabrous); (C) male flower of *P. lasiocarpa* (floral disc parted); (D) female flower of *P. lasiocarpa* (floral disc parted, ovary pannose); (E) capsule of *P. cathayana* (floral disc persistent, pericarp glabrous); (F) capsule of *P. lasiocarpa* (floral disc deciduous, pericarp tomentose); (G) female flower of *P. gonggaensis* (floral disc parted, ovary partly pannose); (H) fruiting branch of *P. cathayana* (leaf abaxially glabrous, base rounded or subcordate); (I) leaf of *P. lasiocarpa* (base deeply cordate); (J) young leaf of *P. lasiocarpa* (abaxially tomentose); (K) young leaf of *P. gonggaensis* (abaxially glabrous, base subcordate).

**Figure 5.** Lectotype of *Populus gonggaensis* N. Chao & J.R. He.

**Table S1.** Information of taxa that were not used for phylogenetic analysis in *Populus* subg. *Tacamahaca*.

**Figure S1.** Species tree of 57 samples of the genus *Populus* constructed by IQ-TREE, based on high-quality SNPs data with an outgroup of *P. euphratica*, using the sliding window method. Pie charts represent the proportion of gene trees that support that specific node. Blue represents gene trees agreeing with the species tree, green those that agree with the main alternative topology, red those that agree with all remaining alternative topologies and grey the proportion of uninformative trees. The numbers on the branches represent the number of concordant gene trees (top) and the number of conflicting trees (bottom).

**Figure S2.** Syntypes of *Populus gonggaensis* N. Chao & J.R. He.

**Table 1. Summary of the statistics of genome resequencing data for 57 individuals of 22 species and one outgroup.**

| **Species** | **Individual** | **Location** | **BioSample ID** | **Vouchers** | **Barcodes or sources** |
| --- | --- | --- | --- | --- | --- |
| *Populus gonggaensis* | *Populus gonggaensis* | Kangding, Sichuan, China | SAMN33060399 | No.4207, Jiaren He et Neng Z | - |
| *Populus heterophylla* | *Populus heterophylla*_1^#^ | Illinois, USA | SAMN17141192 | - | Wang et al. (2022) / NCBI |
| *Populus heterophylla* | *Populus heterophylla*_2 | South Carolina, USA | SAMN33178951 | - | - |
| *Populus heterophylla* | *Populus heterophylla*_3^#^ | New York, USA | SAMN17141193 | - | Wang et al. 2022 / NCBI |
| *Populus heterophylla* | *Populus heterophylla*_4 | Tennessee, Montgomery, USA | SAMN33178952 | - | - |
| *Populus glauca* | *Populus glauca*_1^#^ | Yadong, Xizang, China | SAMN17141151 | - | Wang et al. 2022 / NCBI |
| *Populus glauca* | *Populus glauca*_2^#^ | Ankang, Shaanxi, China | SAMN17141152 | - | Wang et al. 2022 / NCBI |
| *Populus glauca* | *Populus glauca*_3 | Weixi, Yunnan, China | SAMN33178953 | - | - |
| *Populus rockii* | *Populus rockii*_1^#^ | Foping, Shaanxi, China | SAMN17141156 | - | Wang et al. 2022 / NCBI |
| *Populus rockii* | *Populus rockii*_2^#^ | Wenxian, Gansu, China | SAMN17141184 | - | Wang et al. 2022 / NCBI |
| *Populus rockii* | *Populus rockii_3^#^* | Zhen‘an, Shaanxi, China | SAMN17141174 | - | Wang et al. 2022 / NCBI |
| *Populus rockii* | *Populus rockii*_5^#^ | Zhong-Tiao Mountains, Shanxi, China | SAMN17141129 | - | Wang et al. 2022 / NCBI |
| *Populus szechuanica* | *Populus szechuanica* 1^#^ | Yunnan, China | SAMN17141140 |  | Wang et al. 2020 / NCBI |
| *Populus szechuanica* | *Populus szechuanica*_2^#^ | Dali, Yunnan, China | SAMN17141153 | - | Wang et al. 2022 / NCBI |
| *Populus szechuanica* | *Populus szechuanica*_3^#^ | Ebian, Sichuan, China | SAMN17141130 | - | Wang et al. 2022 / NCBI |
| *Populus haoana* | *Populus haoana*_1^#^ | Yunnan, China | SAMN17141167 | - | Wang et al. 2022 / NCBI |
| *Populus haoana* | *Populus haoana*_2^#^ | Yunnan, China | SAMN17141185 | - | Wang et al. 2022 / NCBI |
| *Populus haoana* | *Populus haoana*_3 | Gongshan, Yunnan, China | SAMN33178949 | - | - |
| *Populus laurifolia* | *Populus laurifolia*_1^#^ | Xinjiang, China | SAMN17141138 | - | Wang et al. 2022 / NCBI |
| *Populus laurifolia* | *Populus laurifolia*_2^#^ | Xinjiang, China | SAMN17141118 | - | Wang et al. 2022 / NCBI |
| *Populus laurifolia* | *Populus laurifolia*_3^#^ | Khunjerab National Park, Pakistan | SAMN17141139 | - | Wang et al. 2022 / NCBI |
| *Populus laurifolia* | *Populus laurifolia*_4^#^ | Aketao, Xingjiang, China | SAMN17141159 | - | Wang et al. 2022 / NCBI |
| *Populus cathayana* | *Populus cathayana*_1^#^ | Shannxi, China | SAMN17141127 | - | Wang et al. 2022 / NCBI |
| *Populus cathayana* | *Populus cathayana*_2^#^ | Hebei, China | SAMN17141163 | - | Wang et al. 2022 / NCBI |
| *Populus cathayana* | *Populus cathayana*_3^#^ | Sichuan, China | SAMN17141172 | - | Wang et al. 2022 / NCBI |
| *Populus cathayana* | *Populus cathayana*_4 | Kangding, Sichuan, China | SAMN33060396 | I-3103, Ce Shang | BJFC00091507 |
| *Populus koreana* | *Populus koreana*_1^#^ | Jilin, China | SAMN17141148 | - | Wang et al. 2022 / NCBI |
| *Populus koreana* | *Populus koreana*_2^#^ | Heilongjiang, China | SAMN17141149 | - | Wang et al. 2022 / NCBI |
| *Populus koreana* | *Populus koreana*_3^#^ | Chifeng, Nei Mongol, China | SAMN17141162 | - | Wang et al. 2022 / NCBI |
| *Populus pseudoglauca* | *Populus pseudoglauca*_1^#^ | Mainling, Xizang, China | SAMN17141168 | - | Wang et al. 2022 / NCBI |
| *Populus pseudoglauca* | *Populus pseudoglauca*_2^#^ | Mainling, Xizang, China | SAMN17141136 | - | Wang et al. 2022 / NCBI |
| *Populus ciliata* | *Populus ciliata*^#^ | Mainling, Xizang, China | SAMN17141175 | - | Wang et al. 2022 / NCBI |
| *Populus xiangchengensis* | *Populus xiangchengensis*_1^#^ | Kangding, Sichuan, China | SAMN17141168 | - | Wang et al. 2022 / NCBI |
| *Populus xiangchengensis* | *Populus xiangchengensis*_2^#^ | Markam, Xizang, China | SAMN17141136 | - | Wang et al. 2022 / NCBI |
| *Populus xiangchengensis* | *Populus xiangchengensis*_3^#^ | Xiangcheng, Sichuan, China | SAMN17141128 | - | Wang et al. 2022 / NCBI |
| *Populus xiangchengensis* | *Populus xiangchengensis*_4^#^ | Gongshan, Yunnan, China | SAMN17141166 | - | Wang et al. 2022 / NCBI |
| *Populus xiangchengensis* | *Populus xiangchengensis*_5 | Kangding, Sichuan, China | SAMN33178950 | - | BJFC00091508 |
| *Populus afghanica* | *Populus afghanica*^#^ | Xinjiang, China | SAMN17141165 | - | Wang et al. 2022 / NCBI |
| *Populus iliensis* | *Populus iliensis*^#^ | Xinjiang, China | SAMN17141158 | - | Wang et al. 2022 / NCBI |
| *Populus kangdingensis* | *Populus kangdingensis*^#^ | Sichuan, China | SAMN17141132 | - | Wang et al. 2022 / NCBI |
| *Populus lasiocarpa* | *Populus lasiocarpa*_1^#^ | Sichuan, China | SAMN17141164 | - | Wang et al. 2022 / NCBI |
| *Populus lasiocarpa* | *Populus lasiocarpa*_2^#^ | Hubei, China | SAMN17141170 | - | Wang et al. 2022 / NCBI |
| *Populus lasiocarpa* | *Populus lasiocarpa*_3* | - | SAMC065352 | - | Wang et al. 2020 / GSA |
| *Populus lasiocarpa* | *Populus lasiocarpa*_4* | - | SAMC065353 | - | Wang et al. 2020 / GSA |
| *Populus lasiocarpa* | *Populus lasiocarpa*_5* | - | SAMC065354 | - | Wang et al. 2020 / GSA |
| *Populus nigra* | *Populus nigra*_1^#^ | Shannxi, China | SAMN17141114 | - | Wang et al. 2022 / NCBI |
| *Populus nigra* | *Populus nigra*_2^#^ | Xinjiang, China | SAMN17141142 | - | Wang et al. 2022 / NCBI |
| *Populus qamdoensis* | *Populus qamdoensis*^#^ | Qamdo, Xizang, China | SAMN17141117 | - | Wang et al. 2022 / NCBI |
| *Populus simonii* | *Populus simonii*_1^#^ | Taibai, Shaanxi, China | SAMN17141123 | - | Wang et al. 2022 / NCBI |
| *Populus simonii* | *Populus simonii*_2^#^ | Aba, Sichuan, China | SAMN17141124 | - | Wang et al. 2022 / NCBI |
| *Populus trinervis* | *Populus trinervis*_3^#^ | Wenxian, Gansu, China | SAMN17141125 | - | Wang et al. 2022 / NCBI |
| *Populus trinervis* | *Populus trinervis*_4^#^ | Wuwei, Gansu, China | SAMN17141126 | - | Wang et al. 2022 / NCBI |
| *Populus trinervis* | *Populus trinervis*_1 | Kangding, Sichuan, China | SAMN33060397 | I-3107, Ce Shang | BJFC00091509 |
| *Populus trinervis* | *Populus trinervis*_2 | Kangding, Sichuan, China | SAMN33060398 | I-3114, Ce Shang | BJFC00091510 |
| *Populus yunnanensis* | *Populus yunnanensis*_1^#^ | Lijiang, Yunnan, China | SAMN17141154 | - | Wang et al. 2022 / NCBI |
| *Populus yunnanensis* | *Populus yunnanensis*_2^#^ | Kunming, Yunnan, China | SAMN17141169 | - | Wang et al. 2022 / NCBI |
| *Populus euphratica* | *Populus euphratica*^#^ | Qinghai, China | SAMN17141146 |  | Wang et al. 2020 / NCBI |

**Note**: The individuals for which genome sequences were downloaded from the Genome Sequence Archive (GSA) are marked by asterisks, while those downloaded from the NCBI are marked by the hash (#) sign and the rest are data from these two papers (Wang et al. 2020, 2022). Samples without symbol markings are from new data in this study.

**Table 2.** Morphological comparison of *P. gonggaensis* with *P. cathayana* and *P. lasiocarpa*.

| **Traits** | ***P. gonggaensis*** | ***P. cathayana*** | ***P. lasiocarpa*** |
| --- | --- | --- | --- |
| Petiole | Pubescent. | Pilose. | Glabrous. |
| Leaf blade | Ovate; adaxially glabrous; abaxially glabrous when young; base subcordate; apex acuminate. | Ovate, elliptic-ovate, elliptic or narrowly ovate; adaxially glabrous; abaxially glabrous; base rounded or subcordate; apex acuminate or mucronate. | Ovate; adaxially glabrous; abaxially tomentose when young, and then tomentose along veins; base deeply cordate; apex acuminate. |
| Male flower | – | Floral disc entire. | Floral disc parted. |
| Female flower | Floral disc parted, ovary partly pannose. | Floral disc entire; ovary glabrous. | Floral disc parted; ovary pannose. |
| Capsule | Ovoid, pilose, 3-valved; pedicels 1 mm long, glabrous. | Floral disc persistent, pericarp glabrous. | Floral disc deciduous, pericarp tomentose. |

**Figure 1.** (A) ML tree of 57 samples of the genus *Populus* reconstructed by IQ-TREE, based on 4,790,248 high-quality SNPs with an outgroup of *P. euphratica*; (B) ML tree of 56 samples (*P. gonggaensis* is deleted) of the genus *Populus* reconstructed by IQ-TREE, based on 4,790,248 high-quality SNPs with an outgroup of *P. euphratica*.


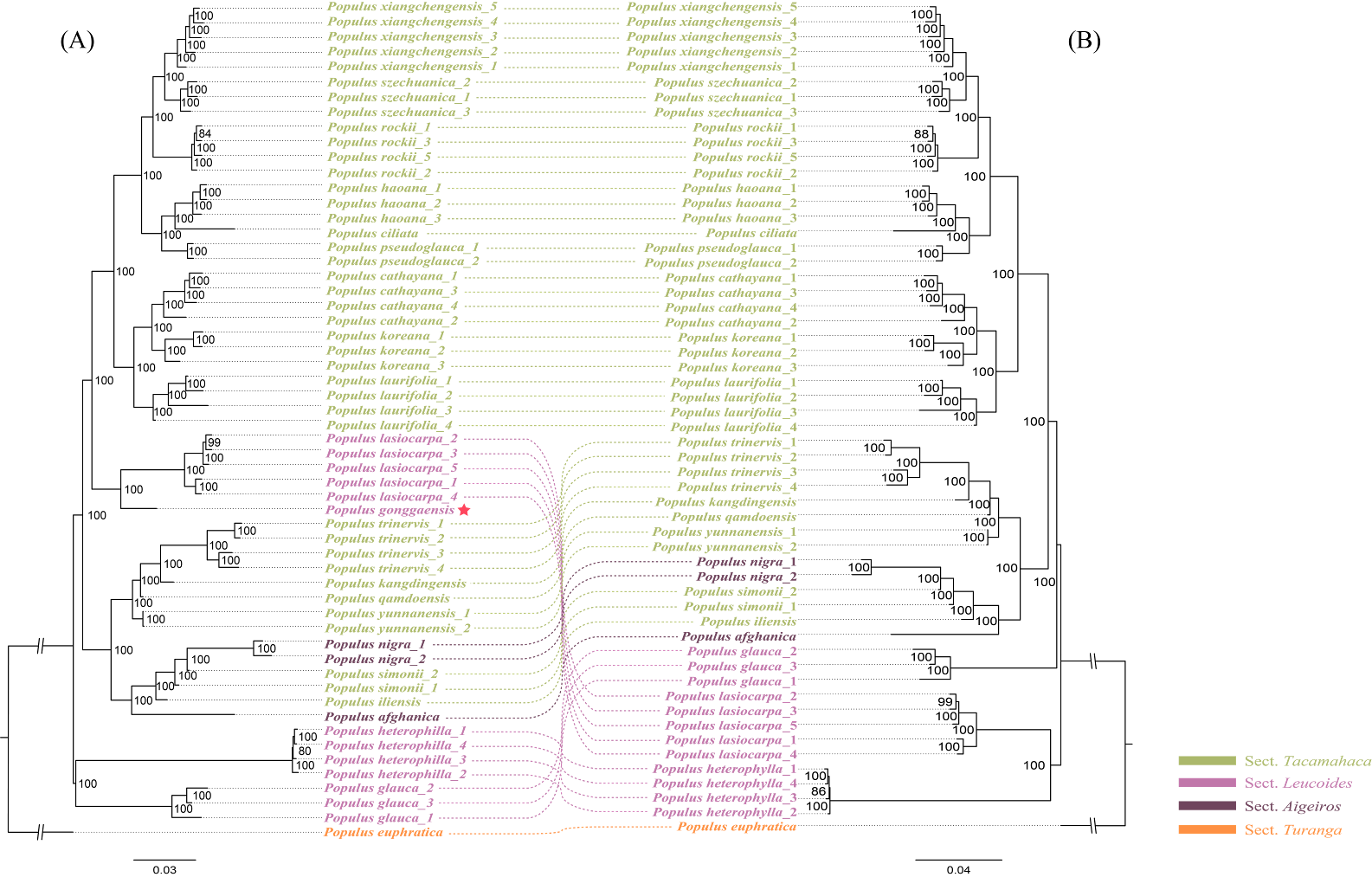


**Figure 2.** Principal Component Analysis (PCA), based on genetic distance using SNPs data. (A) All 57 samples representing 22 species; (B) 37 samples representing 12 species, including the taxa within the red rectangle in (A); (C) Plotted by adding PC3 (Z-axis) to (B). Points inside red circle represent *Populus lasiocarpa*, while those inside blue circle represent *P. cathayana* and *P. koreana*.


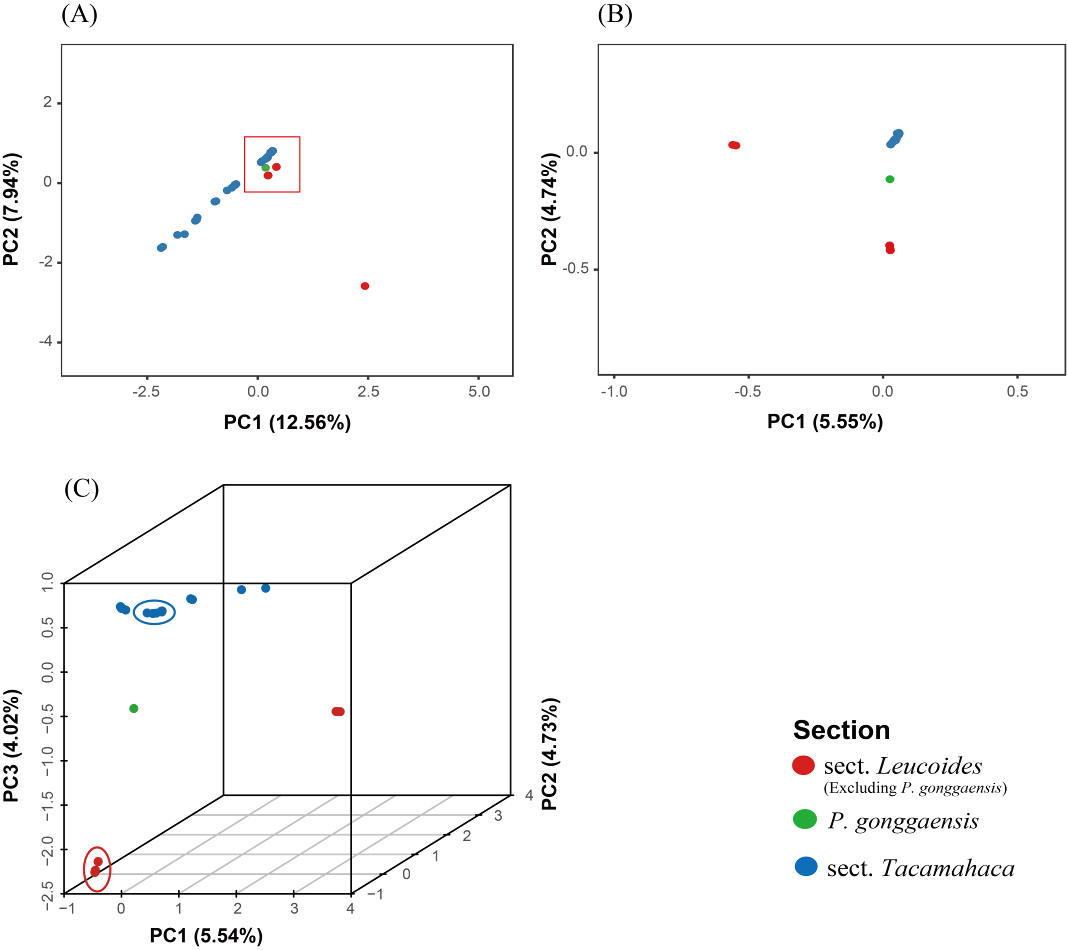


**Figure 3.** Hybrid introgression analysis, using SNP matrix, of *Populus* species. (A) The result of ABBA-BABA statistics which measured gene flow amongst 12 species when P3 = *P. gonggaensis*. When D > 0 and the D value is further away from 0, it indicates that a gene swap or hybridisation event is more likely to have occurred, which means the genes of P3 are more likely to swap with those of P1 or P2; (B) Population structure analysis for 12 species of *Populus* (K = 9). Each coloured bar represents one individual and coloured segments represent proportions of ancestral components. The number of individuals and species names for each lineage are shown at the bottom.


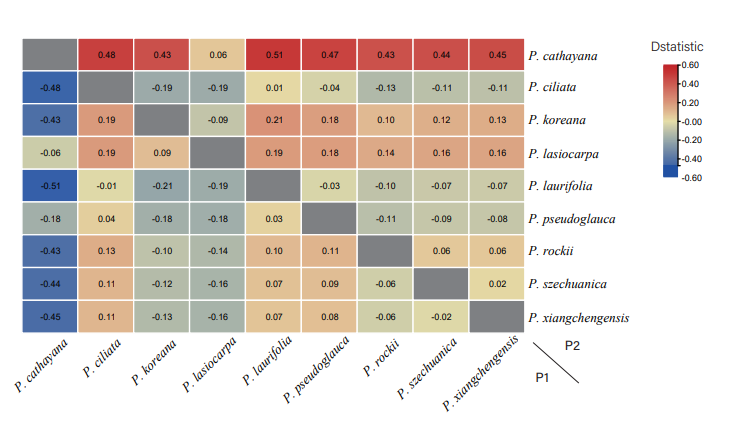
(A)


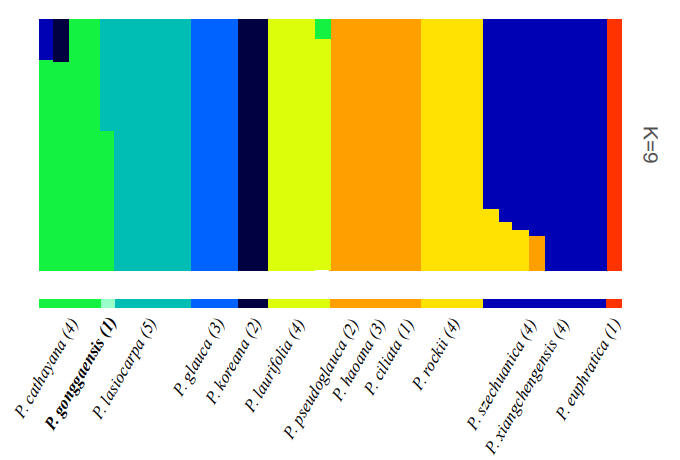
(B)

**Figure 4.** Morphological comparison of *Populus gonggaensis* with *P. cathayana* and *P. lasiocarpa*. (A) male flower of *P. cathayana* (floral disc entire); (B) female flower of *P. cathayana* (floral disc entire, ovary glabrous); (C) male flower of *P. lasiocarpa* (floral disc parted); (D) female flower of *P. lasiocarpa* (floral disc parted, ovary pannose); (E) capsule of *P. cathayana* (floral disc persistent, pericarp glabrous); (F) capsule of *P. lasiocarpa* (floral disc deciduous, pericarp tomentose); (G) female flower of *P. gonggaensis* (floral disc parted, ovary partly pannose); (H) fruiting branch of *P. cathayana* (leaf abaxially glabrous, base rounded or subcordate); (I) leaf of *P. lasiocarpa* (base deeply cordate); (J) young leaf of *P. lasiocarpa* (abaxially tomentose); (K) young leaf of *P. gonggaensis* (abaxially glabrous, base subcordate).


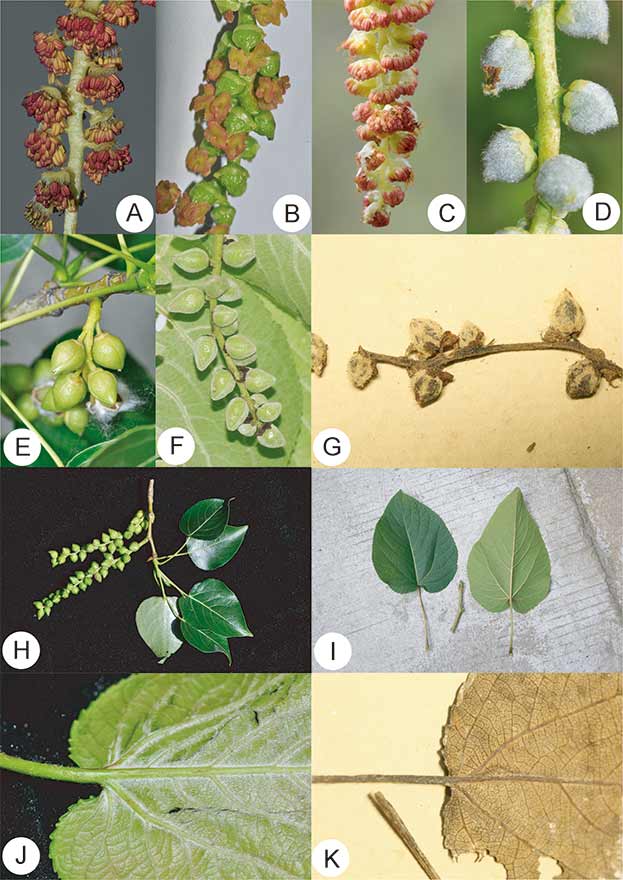


**Figure 5.** Lectotype of *Populus gonggaensis* N. Chao & J.R. He.


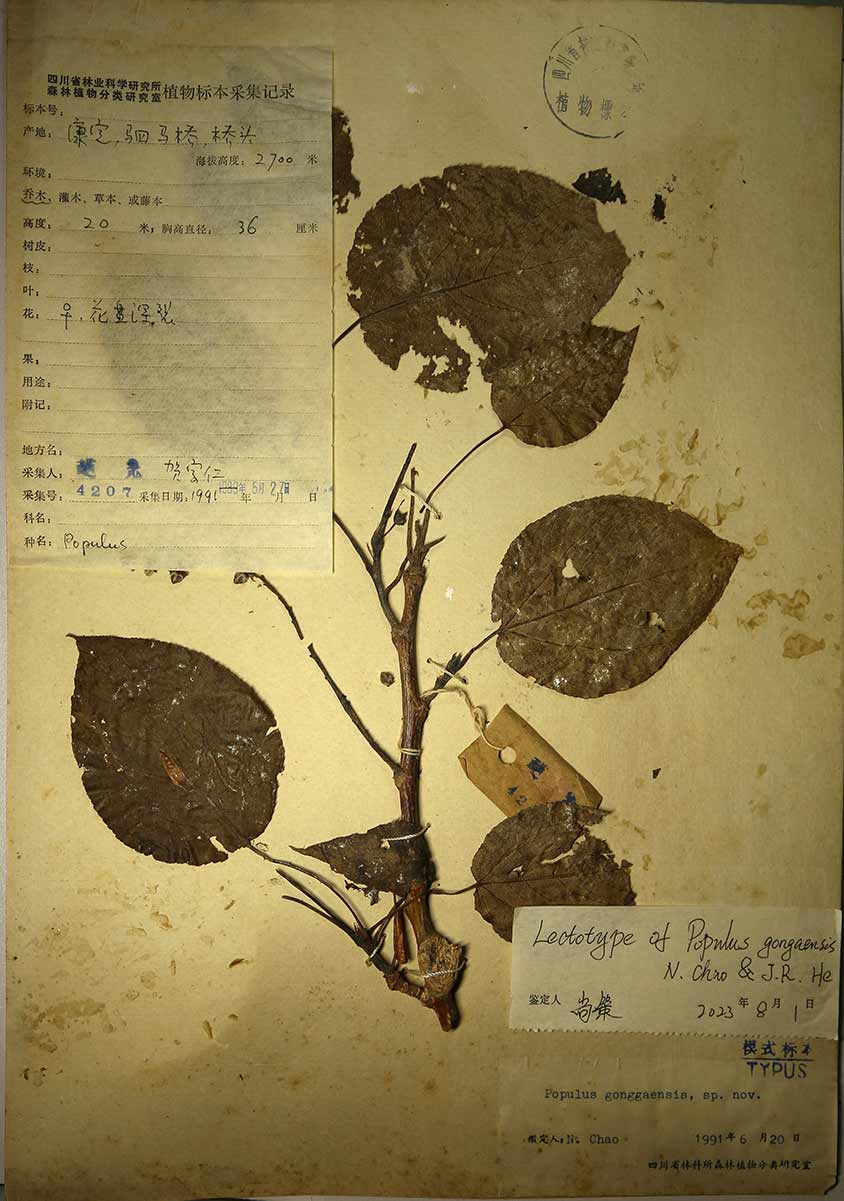


**Table S1. Information of taxa that were not used for phylogenetic analysis in *Populus* subg. *Tacamahaca.***

| **Subclade** | **Scientific name** | **Section** | **Distribution** |
| --- | --- | --- | --- |
| Subclade I | *Populus* × *jrtyschensis* | *Aigeiros* | Xinjiang |
|  | *Populus platyphylla* | *Tacamahaca* | Hebei, Inner Mongolia, Shanxi |
|  | *Populus yunnanensis* var. *microphylla* | *Tacamahaca* | Yunnan |
| Subclade IV | *Populus mainlingensis* | *Tacamahaca* | Tibet |
|  | *Populus szechuanica* var. *tibetica* | *Tacamahaca* | Sichuan, Tibet |
|  | *Populus yatungensis* | *Tacamahaca* | Tibet |
| Subclade V | *Populus intramongolica* | *Tacamahaca* | Hebei, Inner Mongolia, Shanxi |
|  | *Populus maximowiczii* | *Tacamahaca* | Heilongjiang, Inner Mongolia, Jilin, Liaoning, Hebei, Shaanxi |
|  | *Populus pamirica* | *Tacamahaca* | Xinjiang |
|  | *Populus pseudomaximowiczii* | *Tacamahaca* | Hebei |
|  | *Populus shanxiensis* | *Tacamahaca* | Shanxi |
|  | *Populus suaveolens* | *Tacamahaca* | Inner Mongolia, Shaanxi |
|  | *Populus talassica* | *Tacamahaca* | Xinjiang |
|  | *Populus ussuriensis* | *Tacamahaca* | Heilongjiang, Jilin, Liaoning |

**Figure S1.** Species tree of 57 samples of the genus *Populus* constructed by IQ-TREE, based on high-quality SNPs data with an outgroup of *P. euphratica*, using the sliding window method. Pie charts represent the proportion of windows that support that specific node. Blue represents windows concordant with the species tree, green those that agree with the main alternative topology, red those that agree with all remaining alternative topologies and grey the proportion of uninformative
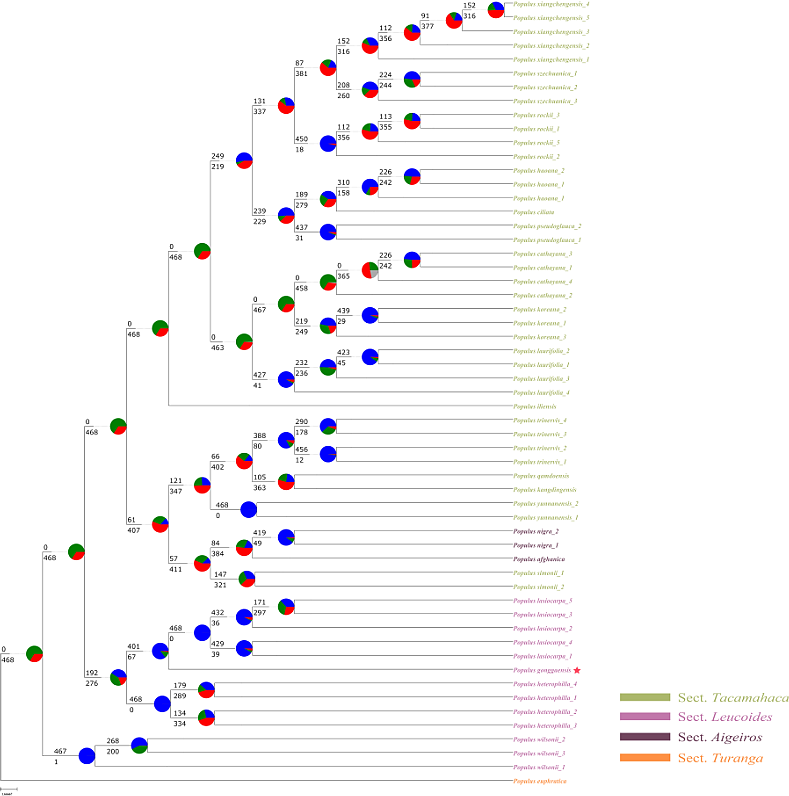
trees. The numbers on the branches represent the number of windows that support the species tree (top) and the number of conflicting trees (bottom).

**Figure S2.** Syntypes of *Populus gonggaensis* N. Chao & J.R. He.
