# Supplementary material for: ﻿Phylogenomics reveal Populusgonggaensis as a hybrid between P.lasiocarpa and P.cathayana (Salicaceae)
Source: PhytoKeys. 2024 Jan 23;237:161–77. doi: 10.3897/phytokeys.237.103012 (PMC10829108; doi:10.3897/phytokeys.237.103012)
Supplement: Supplementary material 2 — Species tree of 57 samples of the genus Populus constructed by IQ-TREE, based on high-quality SNPs data with an outgroup of P.euphratica, using the sliding window method [file phytokeys-237-161_article-103012__-s002.pdf]

Since the molecular data was extracted from specimen collected long ago, the quality of sequencing sample is somewhat low. Thus, it is impossible to use single-copy genes to construct a species tree. Instead, we used the sliding window method to construct a phylogenetic tree based on SNP dataset. We used ASTRAL-III v.5.15.1 to infer a species tree from the whole SNPs dataset based on the coalescent theory, setting the window size to 50k and using all gene trees as input data.

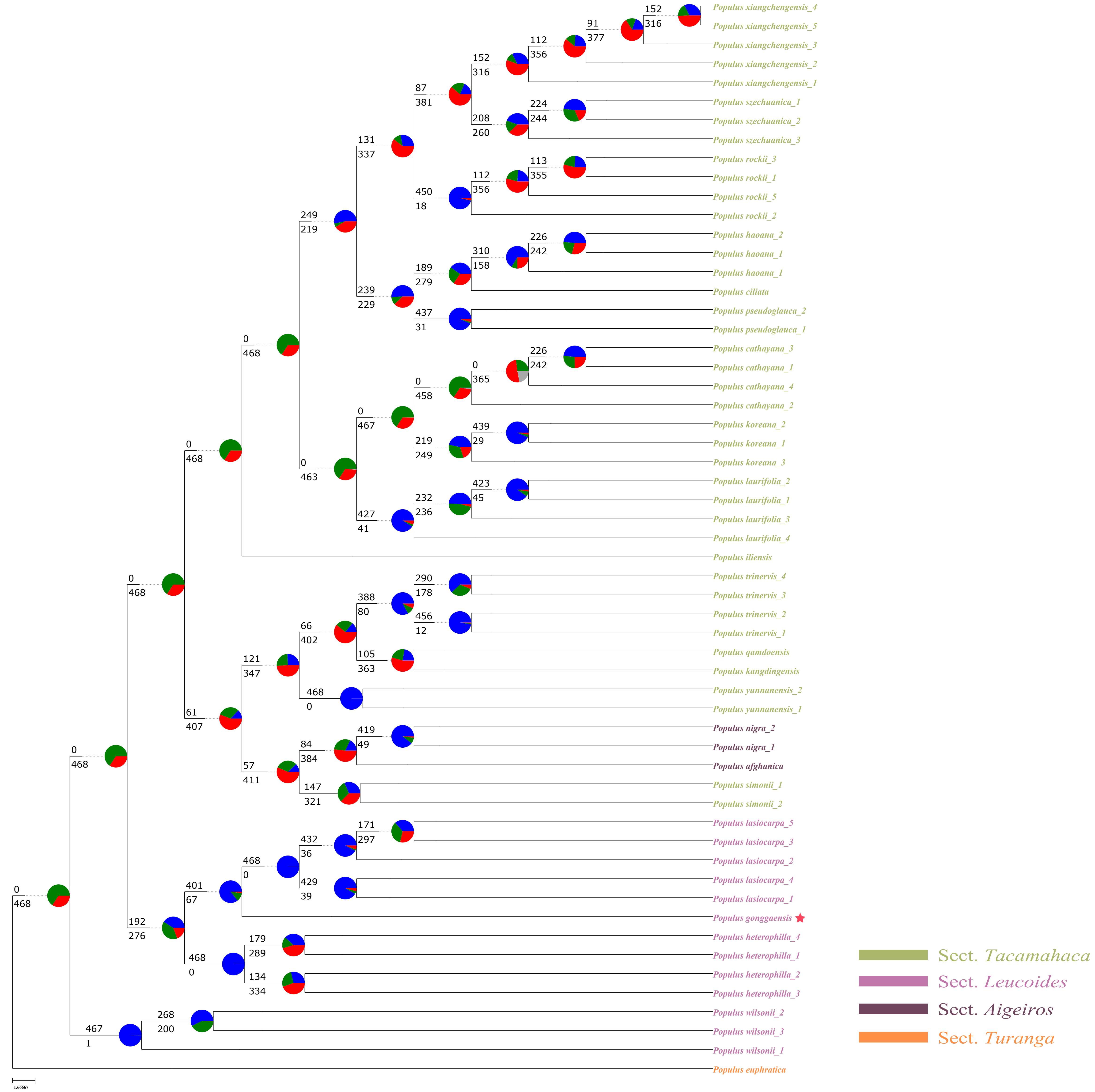

Populus xiangchengensis_4
Populus xiangchengensis_5
Populus xiangchengensis_3
Populus xiangchengensis_2
Populus xiangchengensis_1
Populus szechuanica_1
Populus szechuanica_2
Populus szechuanica_3
Populus rockii_3
Populus rockii_1
Populus rockii_5
Populus rockii_2
Populus haoana_2
Populus haoana_1
Populus haoana_1
Populus ciliata
Populus pseudoglauca_2
Populus pseudoglauca_1
Populus cathayana_3
Populus cathayana_1
Populus cathayana_4
Populus cathayana_2
Populus koreana_2
Populus koreana_1
Populus koreana_3
Populus laurifolia_2
Populus laurifolia_1
Populus laurifolia_3
Populus laurifolia_4
Populus iliensis
Populus trinervis_4
Populus trinervis_3
Populus trinervis_2
Populus trinervis_1
Populus qamdoensis
Populus kangdingensis
Populus yunnanensis_2
Populus yunnanensis_1
Populus nigra_2
Populus nigra_1
Populus afghanica
Populus simonii_1
Populus simonii_2
Populus lasiocarpa_5
Populus lasiocarpa_3
Populus lasiocarpa_2
Populus lasiocarpa_4
Populus lasiocarpa_1
Populus gonggaensis
Populus heterophilla_4
Populus heterophilla_1
Populus heterophilla_2
Populus heterophilla_3
Populus wilsonii_2
Populus wilsonii_3
Populus wilsonii_1
Populus euphratica
1.66667
Sect. Tacamahaca
Sect. Leucoides
Sect. Aigeiros
Sect. Turanga
