# Supplementary figures and images for: ﻿Phylogenomics reveal Populusgonggaensis as a hybrid between P.lasiocarpa and P.cathayana (Salicaceae)
Source: PhytoKeys. 2024 Jan 23;237:161–77. doi: 10.3897/phytokeys.237.103012 (PMC10829108; doi:10.3897/phytokeys.237.103012)

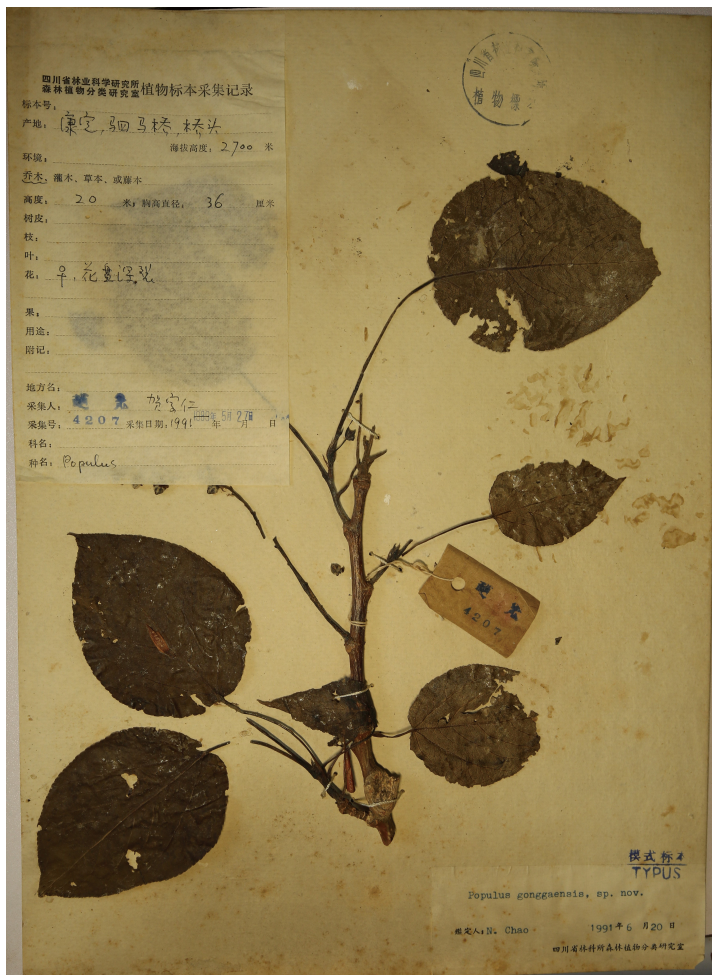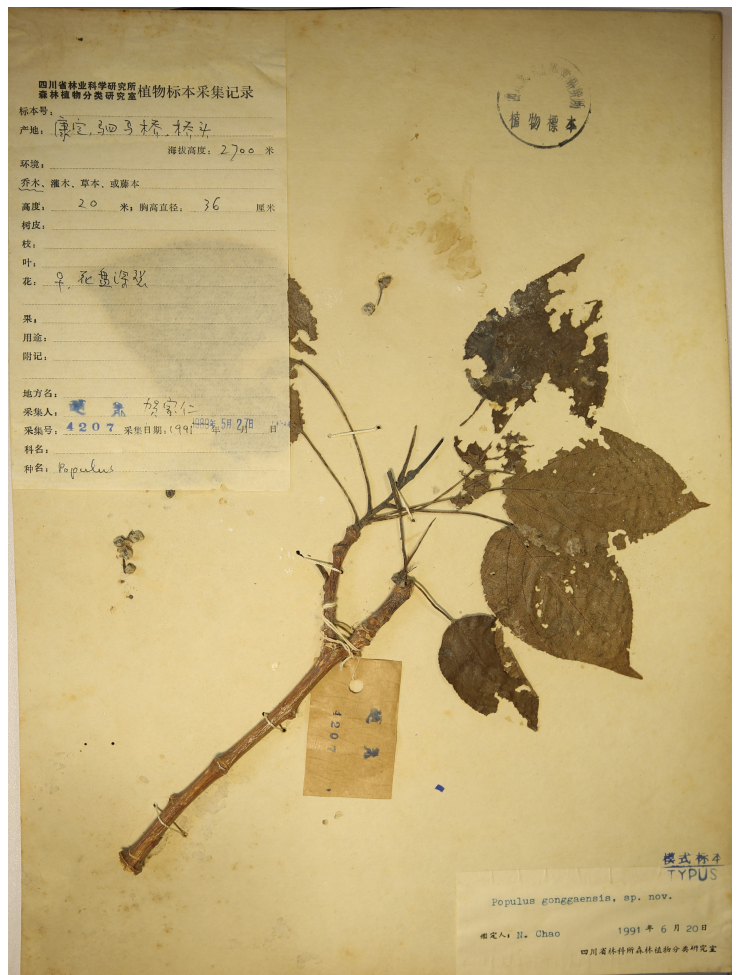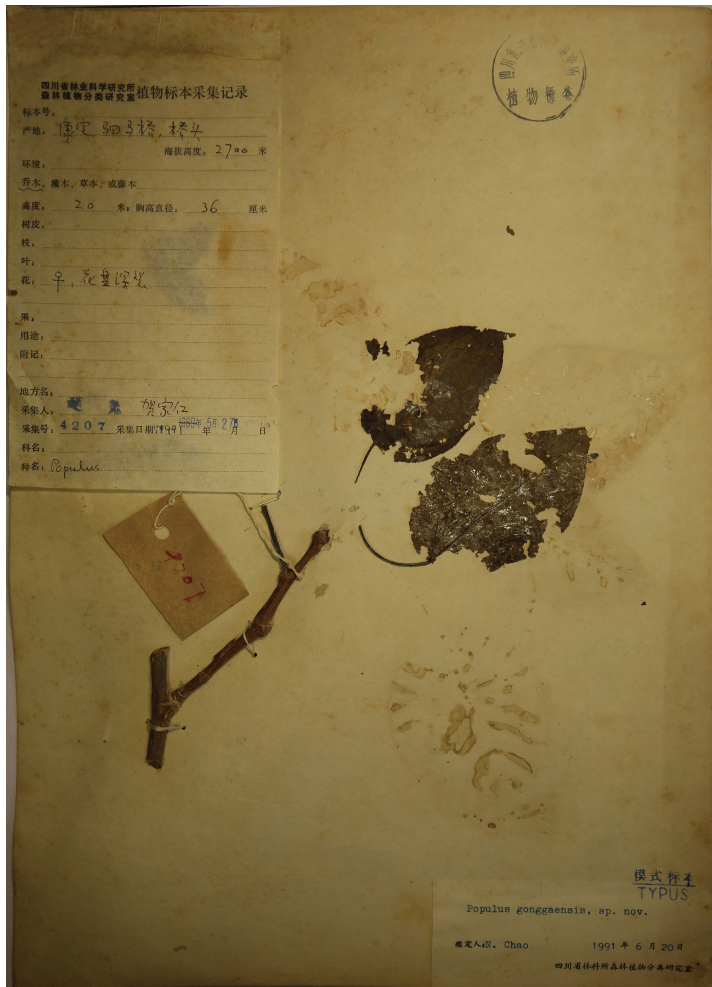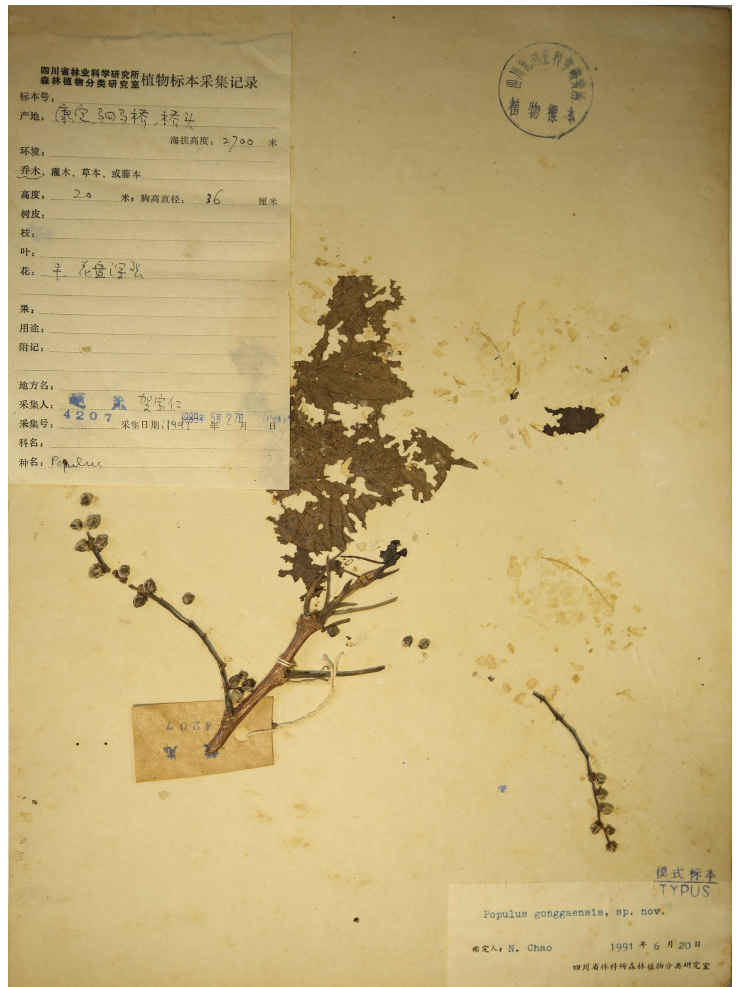

Supplement: Supplementary material 3 — Syntypes of Populusgonggaensis N. Chao & J.R. He [file phytokeys-237-161_article-103012__-s003.pdf]
